# Site-specific *N*-glycosylation analysis of animal cell culture-derived Zika virus proteins

**DOI:** 10.1038/s41598-021-84682-z

**Published:** 2021-03-04

**Authors:** Alexander Pralow, Alexander Nikolay, Arnaud Leon, Yvonne Genzel, Erdmann Rapp, Udo Reichl

**Affiliations:** 1 Bioprocess Engineering Group, Max Planck Institute for Dynamics of Complex Technical Systems, Magdeburg, Germany; 2 Valneva SE, Saint-Herblain, France; 3 glyXera GmbH, Magdeburg, Germany; 4 Chair of Bioprocess Engineering, Otto von Guericke University, Magdeburg, Germany

**Keywords:** Biochemistry, Biotechnology, Molecular biology, Structural biology, Biomarkers, Diseases

## Abstract

Here, we present for the first time, a site-specific *N*-glycosylation analysis of proteins from a Brazilian Zika virus (ZIKV) strain. The virus was propagated with high yield in an embryo-derived stem cell line (EB66, Valneva SE), and concentrated by g-force step-gradient centrifugation. Subsequently, the sample was proteolytically digested with different enzymes, measured via a LC–MS/MS-based workflow, and analyzed in a semi-automated way using the in-house developed glyXtool^MS^ software. The viral non-structural protein 1 (NS1) was glycosylated exclusively with high-mannose structures on both potential *N*-glycosylation sites. In case of the viral envelope (E) protein, no specific *N*-glycans could be identified with this method. Nevertheless, *N*-glycosylation could be proved by enzymatic de-*N*-glycosylation with PNGase F, resulting in a strong MS-signal of the former glycopeptide with deamidated asparagine at the potential *N*-glycosylation site N444. This confirmed that this site of the ZIKV E protein is highly *N*-glycosylated but with very high micro-heterogeneity. Our study clearly demonstrates the progress made towards site-specific *N*-glycosylation analysis of viral proteins, i.e. for Brazilian ZIKV. It allows to better characterize viral isolates, and to monitor glycosylation of major antigens. The method established can be applied for detailed studies regarding the impact of protein glycosylation on antigenicity and human pathogenicity of many viruses including influenza virus, HIV and corona virus.

## Introduction

Zika virus (ZIKV) infection during pregnancy can compromise brain development of the fetus, which can lead to severe congenital microcephaly^1–3^. Furthermore, recent ZIKV outbreaks have been associated with increasing cases of the Guillain–Barré syndrome^4–6^. The outbreak in the Americas launched a tremendous interest in studying ZIKV, starting with the identification and sequencing of ZIKV from amniotic fluid of fetuses showing microcephaly in Brazil^7^, and continuing with various approaches towards antiviral treatment and vaccine development.

The ZIKV genome encodes for three glycoproteins: (1) envelope (E) protein, (2) non-structural protein 1 (NS1) and (3) precursor membrane protein M (prM)^7,8^. The E protein is a surface glycoprotein and is considered as one of the major antigens among flaviviruses for vaccine development. It facilitates viral entry into host cells and is the major target for neutralizing antibodies due to its high expression level in the cell’s envelope^9^. One potential *N*-glycosylation site of the E protein is described, which may however mutate, leading to a loss of the consensus sequence^9^. Its glycosylation plays a major role for infectivity, viral assembly and secretion of the ZIKV^10^. Recently, glycosylation of the ZIKV E protein was demonstrated to correlate with host–virus interaction and pathogenesis^11,12^. The non-structural protein 1 (NS1) has two potential *N*-glycosylation sites. It is crucial for the evasion of the host immune system via inhibiting type 1 interferon production by impeding tank-binding kinase 1 (TBK1) complex formation^13,14^. Finally, the prM protein with its single potential *N*-glycosylation site is important for the assembly of mature virions through cleavage of prM into the membrane (M) protein^15^.

Glycosylation is a non-template driven (co-)post-translational modification whereby complex oligosaccharides (most commonly *N*- and *O*-linked glycans) are enzymatically attached to the proteins at various potential sites^16^. *N*-glycans are linked to the amino group of asparagine according to a specific consensus sequence (NXS/T; X ≠ P). Moreover, *N*-glycans are characterized by a common core-structure GlcNAc_2_Man_3_ (*N*-acetylglucosamine (GlcNAc), mannose (Man)), which can be extended to form complex-, high-mannose- or hybrid-type *N*-glycan structures^17^.

To our knowledge, no site-specific *N*-glycosylation analysis of intact ZIKV proteins was accomplished so far. Recently, Routhu et al. (2019) performed the analysis of released *N*-glycans derived from SDS-gel separated ZIKV E protein (propagated in different cell lines) via MALDI-TOF–MS and lectin microarrays^12^. The authors identified complex-(sialo and asialo) and high-mannose-type *N*-glycans. Glycomic methods, however, harbor the risk of analyzing non-viral *N*-glycans derived from cell culture, contaminating the *N*-glycan profile of the ZIKV glycoproteins if the downstream processing is not efficient enough. Until now, only site-specific *N*-glycosylation analysis can elucidate the three-dimensional glycosylation pattern of glycoproteins.

High-yield cell culture processes for Brazilian ZIKV production^18^ enable a closer look on the glycosylation pattern of the ZIKV proteins by providing enough material for detailed analytical studies. Here, ZIKV glycoproteins were analyzed using nano reversed-phase liquid chromatography (nanoRP-LC) coupled to tandem mass spectrometry (MS/MS), followed by manual and semi-automated analysis of higher-energy collisional dissociation (HCD)-generated glycopeptide fragment ion spectra using glyXtool^MS^^19^, an in-house-developed glycopeptide analysis software.

Regarding early vaccine candidate identification, selection of expression systems and process development, detailed glycosylation studies can be essential to understand and meet quality attributes in terms of antigenicity and immunogenicity.

## Materials and methods

**Figure 1:**
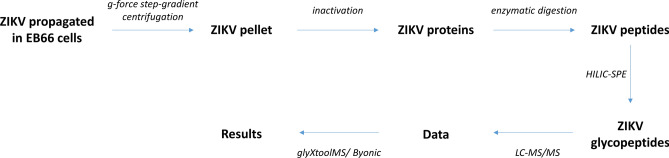
Workflow of the site-specific *N*-glycopeptide analysis of ZIKV proteins.

A workflow depicting the site-specific *N*-glycopeptide analysis of ZIKV proteins is shown in Fig. 1.

Enzymes used for proteomic digestion were trypsin (Trypsin Sequencing Grade Modified, V5111, Promega, Madison, WI, USA) endoproteinase AspN [Flavastacin^20^] (AspN, P8104S, New England Biolabs, Ipswich, MA, USA), proteinase K (A4392, AppliChem, Darmstadt, Germany) and peptide *N*-glycosidase F (PNGase F, P7367, Sigma-Aldrich, Steinheim, Germany). Chemicals for analytics were DL-dithiothreitol (DTT, D5545-5G), iodoacetamide (IAA, I1149-25G), ammonium bicarbonate (ABC, 09830-500G) and acetonitrile (ACN, LC–MS Grade ≥ 99.5%, 34967) (Sigma-Aldrich, Steinheim, Germany), urea (A1049), Tris–HCL (A3452) and calcium chloride (A46899) (AppliChem, Darmstadt, Germany) and trifluoroacetic acid (TFA; 28904, Thermo Fisher Scientific, Waltham, MA, USA). All solvents for LC–MS were LC–MS grade. Chemicals for analytical sample preparation were of highest grade available. All buffers and solutions were prepared with deionized and purified water (dH2O) using a Milli-Q water purification system (18.2 MΩ cm^−1^ at 25 °C, total organic carbon of 3 ppb) from Merck Millipore. For LC–MS solvents, water was further purified using the LC-Pak Polisher from Merck Millipore.

### Cell lines, cell cultivation and virus propagation

A wild-type ZIKV strain was collected during the ZIKV outbreak in Rio de Janeiro state of Brazil in 2015/2016. Therefore, the virus was isolated from whole blood specimens of a polymerase chain reaction (PCR)-positive adult patient during the acute phase of symptoms. The virus was recovered in C6/36 insect cell culture (virus material by kind permission of T. S. Moreno, Fiocruz, Brazil) and a virus seed was generated from the cleared supernatant of infected African green monkey kidney (Vero WHO ECACC) cells.

The virus was sequentially adapted over five passages to the embryonic EB66 suspension cell line (Valneva SE) as described by Nikolay et al.^18^. Therefore, cells were growing in CDM4Avian medium and infected with a multiplicity of infection (MOI) of 0.01. Four days post infection, 70 μL of the cell broth was transferred to the subsequent shake flask. Further virus passages were carried out with 50 μL every 3 days to select for fast-propagating viruses.

The adapted ZIKV was used to infect a high-cell density perfusion culture as described elsewhere^18^. In brief, EB66 cells grew with a cell-specific perfusion rate of 34 pL/cell/day and were infected at 7.3 × 10^7^ cells/mL with MOI 0.001. Three days post infection, a fraction of the bioreactor was harvested at an infectious virus titer of 8.5 × 10^9^ PFU/mL.

### Virus harvesting, purification and inactivation

ZIKV material was harvested from the perfusion bioreactor run and concentrated via g-force step-gradient centrifugation adapted from previous work^21,22^. The cell culture supernatant (30 mL) was centrifuged at 5000 g for 10 min at 4 °C. Further ultracentrifugation of the resulting supernatant was performed on a 7.2 mL sucrose cushion (20% (w/v) sucrose, 25 mM HEPES in PBS) added to an ultra-clear tube (38 mL, Beckman). Therefore, the sucrose cushion was carefully overlaid with 30.8 mL of the supernatant (virus harvest) and centrifuged at 103,745 g for 289 min (swing rotor SW28 and Optima LE-80 K ultracentrifuge (Beckman Coulter)). Finally, the virus pellet was re-conditioned in 200 µL TNE buffer (10 mM Tris, 0.2 M NaCl, 10 mM EDTA in PBS, pH adjusted to 7.4) and inactivated via incubation for 10 min at 56 °C (Thermomixer, Eppendorf) in 50 µL 100 mM Tris–HCL_(aq)_ buffer with 5% (v/v) SDS. Samples were stored at − 80 °C.

### Protein concentration assay

The protein concentration of the purified ZIKV harvest was determined using the QuantIT protein assay (Q33210, Life technologies, Germany), following the assay instructions from the supplier.

### Proteomics and glycopeptide analysis

Proteolytic digestion of 100 µg ZIKV glycoproteins per sample was performed using a modified version of the filter-aided sample preparation (FASP) approach introduced by Wisniewski et al.^23^. The samples were proteolytically digested with trypsin, trypsin followed by flavastacin (sequential digest) or proteinase K. Afterwards, glycopeptide enrichment was performed using a modified version of the hydrophilic interaction liquid chromatography solid-phase extraction (HILIC-SPE) developed by Selman et al.^24,25^. Because of the use of different enzymes (different peptide moieties of a single glycosylation site) in combination with a glycopeptide specific enrichment strategy (depletion of non-glycosylated peptides), the analysis of the glycoproteins macroheterogeneity is not suitable with our workflow. For nanoRP-LC–MS(/MS) measurement, 500 ng enriched glycopeptides (≈ 1 µg/µL) were analyzed on an Ultimate 3000 nanoLC system online coupled to an LTQ Orbitrap Elite hybrid mass spectrometer (both Thermo Fischer Scientific). A comprehensive description of the entire glycoproteomic analysis workflow can be found in Hoffmann et al.^25^.

Manual glycopeptide analysis was performed according to Pralow et al.^20^ and Hoffmann et al.^25^. Semi-automated glycopeptide and automated peptide analysis was performed using glyXtool^MS^ and Byonic (Protein Metrics using UniProtKB A0A024B7W1) according to Pioch et al.^19^ and Hoffmann et al.^25^.

### *N*-Glycan release

ZIKV peptides after tryptic digestion were lyophilized and reconstituted in PBS. For *N*-glycan release, 1 U of PNGase F was added and samples were incubated for 3 h at 37 °C. Afterwards, the sample was centrifuged through a 10 kDa molecular weight cut-off (MWCO) filter (Nanosep Omega with polyethersulfone membrane, PALL Life Sciences) and the flow through was harvested. De-*N*-glycosylated peptides were directed to LC–MS/MS analysis as previously described^20^.

### Graphical illustration

The molecular structure of the E protein and protein M complex was modeled using the protein data bank (PDB) entry number 5h37. To model the molecular structure of NS1, the PDB entry number 5k6k was used. For model processing and design the open source software UCSF Chimera Version 1.10.2 was utilized.

## Results and discussion

MS-based glycoproteomics is the method of choice to identify site-specific glycosylation of proteins. Recently, we demonstrated such an analysis for influenza A virus, elucidating the site-specific glycosylation of the major antigen hemagglutinin (Gränicher et al.^26^). For the glycoproteins of ZIKV, such an analysis is still missing. While the glycosylation of viral antigens is still not a critical quality attribute in vaccine manufacturing (except for recombinantly produced vaccines, e.g. Flublock), we believe such fundamental analysis of complex biologicals can become crucial in the future as glycosylation may mask antigenic sites, stimulate the host immune response and affect vaccine efficacy^27^.

**Figure 2:**
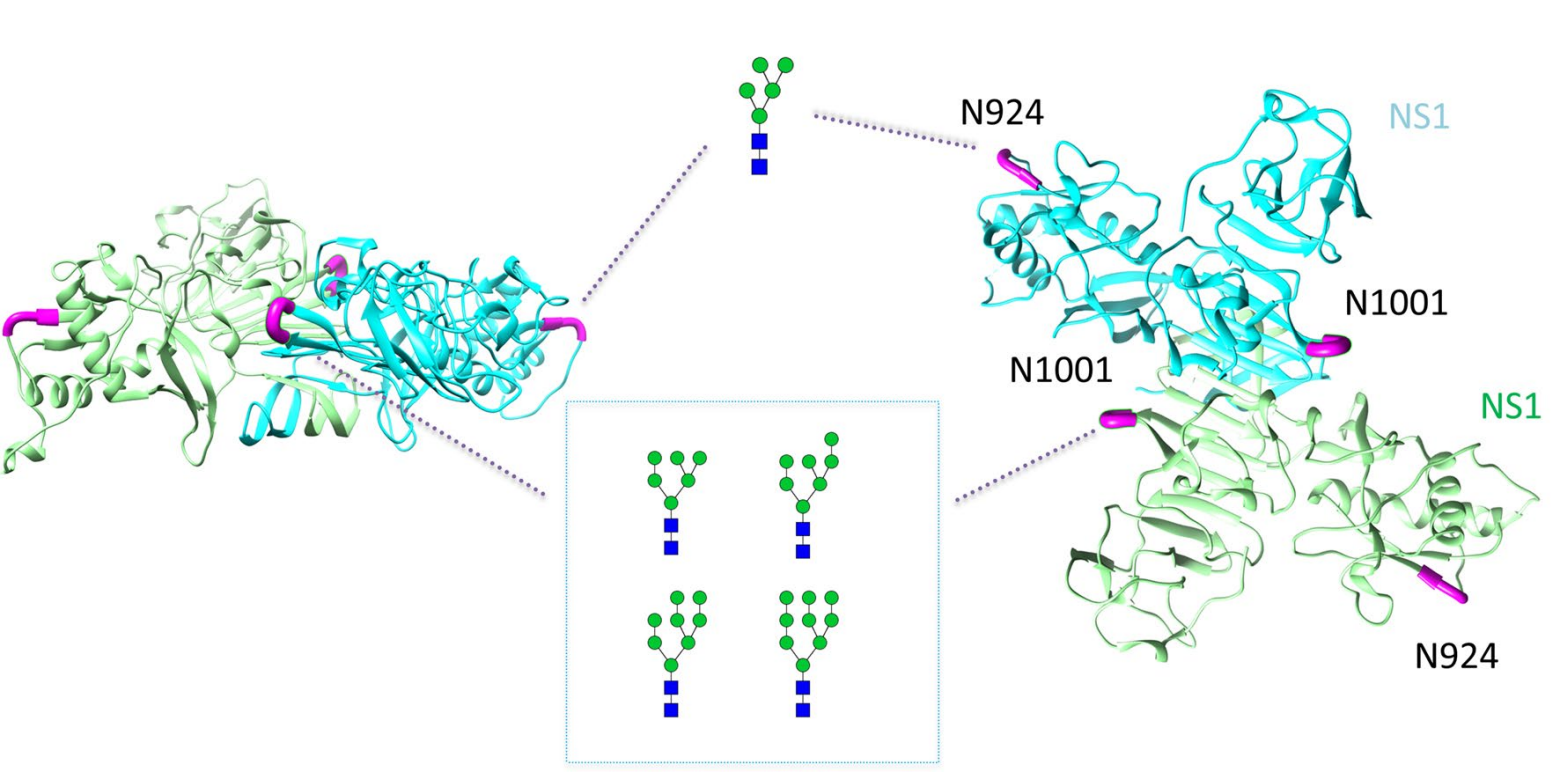
Site-specific *N*-glycosylation of the NS1 protein of ZIKV produced in EB66 cells. The structure of the homodimer NS1 (PDB: 5k6k) is exemplary illustrated. Monomers are visualized using different colors (green and cyan). Potential *N*-glycosylation sites are highlighted in magenta. Annotated *N*-glycans represent only possible *N*-glycan structures based on our results obtained by MS spectra analysis. Symbolic representation of *N*-glycan structures was generated with GlycoWorkbench Version 1.1 following the guideline of Symbol Nomenclature for Graphical Representation of Glycans^31^.

As shown for the three-dimensional structure of the homodimer NS1, the glycoprotein has two potential *N*-glycosylation sites—N924 and N1001 (Fig. 2). Site-specific *N*-glycosylation analysis revealed NS1 to be exclusively *N*-glycosylated with high-mannose-type *N*-glycans on both potential *N*-glycosylation sites, which is in accordance with the literature for other flaviviruses, like dengue virus^28,29^. For dengue virus, the intracellular NS1 dimer is described to occur host cell independent high-mannose-type *N*-glycosylated. Whereby the extracellular NS1 hexamer is described to have complex-type *N*-glycosylation^28^. We assume the results are obtained from intracellular NS1 dimers resulting from cell lysis during long term cultivation or vesicles including NS1 dimers^30^ and might also reflect the glycosylation of the wild ZIKV independent from the host cell line. All annotated MS/MS fragment ion spectra are depicted in the Supplement. At site N924, we could only identify one *N*-glycan referring to Man5. The site N1001 was found to be glycosylated with Man6-Man9 *N*-glycan compositions.

**Figure 3:**
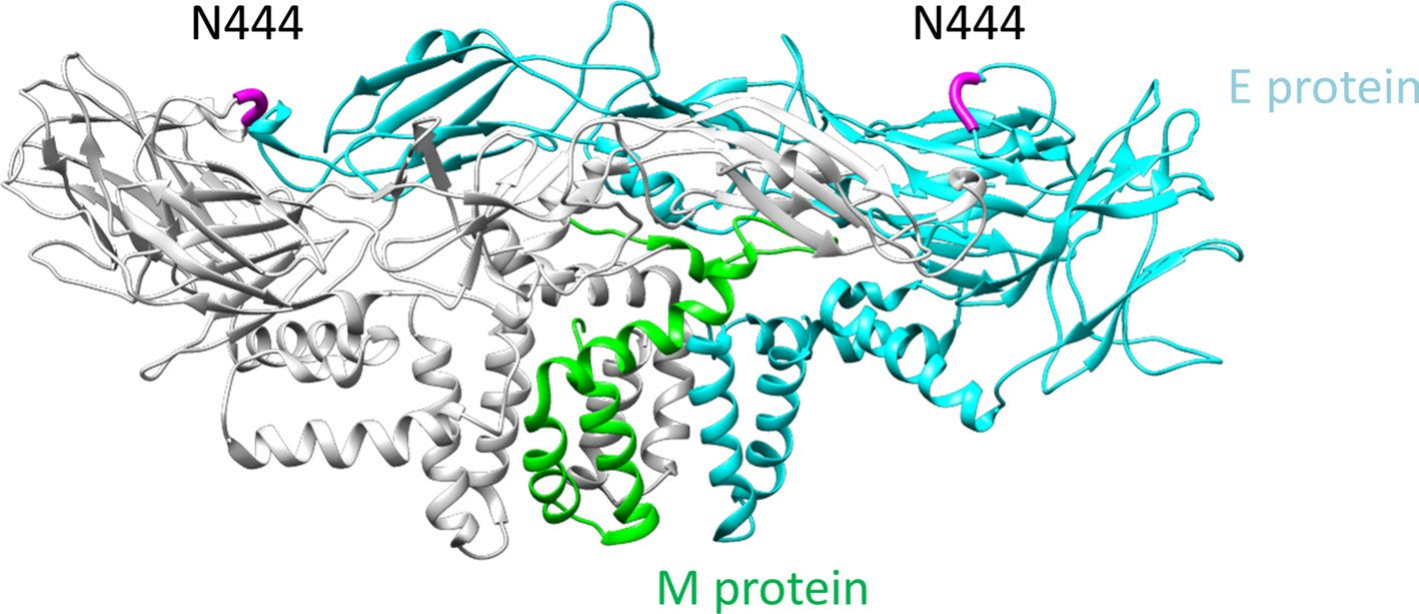
Exemplary illustrated structure of the M/E protein of ZIKV. The structure of the heterodimer protein M and E complex (PDB: 5h37) is illustrated. This complex is formed from an M protein homodimer (monomer visualized in green) and an E protein homodimer (monomer visualized in cyan). The potential *N*-glycosylation site N444 is highlighted in magenta.

The three-dimensional structure of the protein M and E complex (M/E protein) displays a single potential *N*-glycosylation site N444 on top of the head region (Fig. 3). Exposure indicates good accessibility for receptors and antibodies. However, *N*-glycoproteomic analysis did not yield any *N*-glycopeptides of the E protein. Furthermore, although the proteomic analysis did properly cover the sequence of the E protein, we were not able to identify a precursor ion or fragment ion spectrum for the non-glycosylated peptide sequence in the vicinity of N444 of the E protein (see Supplement, Figure 1A). The lab scaled virus purification using g-force step-gradient centrifugation was optimized to the size of the ZIKV. However, traditional proteomics^20^ of the purified virus pellet using mammalian database and ZIKV FASTA resulted in the identification of many host cell proteins co-eluting (see Supplement, Table 1). Therefore, glycoproteomic analysis seems to be the method of choice to identify virus protein glycosylation, because glycomic approaches bear the risk of detecting *N*-glycans from the host cell system, even after gel separation (data not shown).

**Figure 4:**
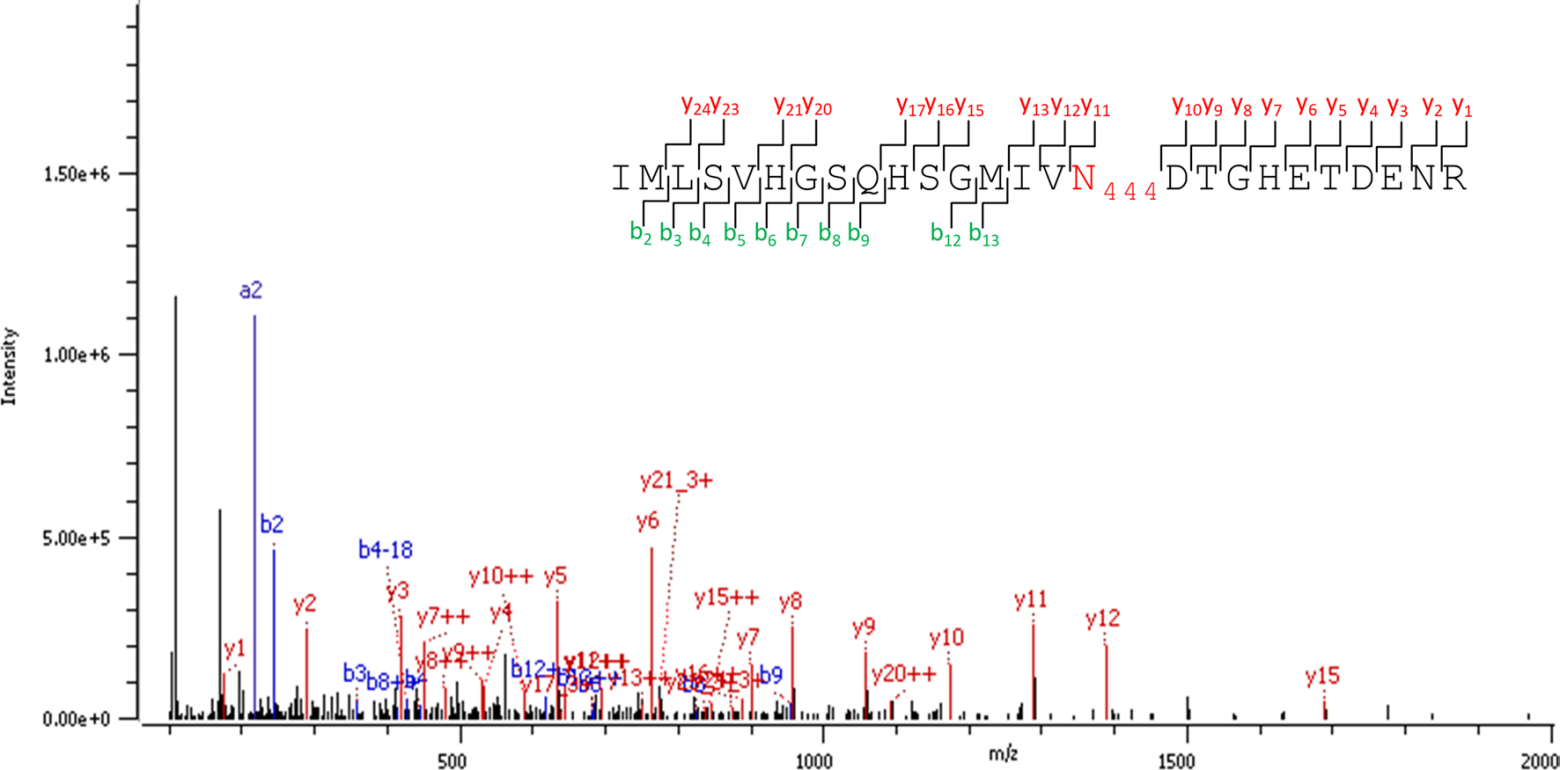
MS/MS fragment ion spectrum of the de-*N*-glycosylated and deamidated *N*-glycopeptide from the ZIKV E protein produced in EB66 cells. The fragment ion spectrum of the tryptic peptide IMLSVHGSQHSGMIVN(deamidated)DTGHETDNR after *N*-glycan release is shown. Diagnostic y-ions (red) and b-ions (blue) are annotated in the ion spectrum and highlighted in the peptide sequence at the upper right corner. The potential *N*-glycosylation site N444 is deamidated (red).

However, to figure out if the E protein is *N*-glycosylated, we performed the de-*N*-glycosylation via PNGase F treatment of our sample, a procedure which is widely accepted to identify *N*-glycoproteins in complex samples^32–35^, as only *N*-glycosylated asparagine (Asn/N) converts to deamidated Asn (= aspartic acid (Asp/D)) with a mass shift of 1 Da. Thus, with this enzymatic de-*N*-glycosylation, all glycoforms of an *N*-glycopeptide converge into one signal of the respective de-*N*-glycosylated peptide with deamidated asparagine, which significantly increases the chance of detection by LC–MS/MS. Doing so (enzymatic *N*-glycan release of the tryptically digested ZIKV proteome by PNGaseF), the various glycoforms of the E protein glycopeptides with N444 resembled into an amino acid sequence with a deamidated N444, i.e. D444. Supplement (Figure 1) shows the sequence coverage of the ZIKV E protein before (A) and after PNGase F treatment (B). After PNGase F treatment, the peptide sequence in the vicinity of the potential *N*-glycosylation site N444 was well covered in comparison to the sample without PNGase F treatment. The corresponding MS/MS fragment ion spectrum of the deamidated tryptic peptide including N444 is shown in Fig. 4. Accordingly, our findings prove that the ZIKV E protein investigated in this study was indeed highly *N*-glycosylated, but with too many different *N*-glycan compositions, resulting in too many low abundant precursor signals to be detected by LC–MS/MS.

Overall, our study clearly demonstrates the progress made towards site-specific *N*-glycosylation analysis of ZIKV proteins. In addition, it might be used for other heavily glycosylated viral proteins, for example the spike S protein of Corona viruses^36^, i.e. SARS-CoV-2^37^. This motivates, especially for viruses with pandemic potential, to investigate the glycosylation of viral proteins more closely, and to associate biological (e.g. virus isolate, virus entry, pathogenicity, antigenicity) and vaccine-related factors (e.g. host cell/expression system, virus seed generation, critical quality attributes) to the glycosylation of the major antigens.

A bottleneck for the site-specific *N*-glycosylation analysis and detection of ZIKV proteins was until now the quantity of virus material provided. Therefore, intensified high-titer virus production processes are necessary to support such complex analyses. In addition, improving the concentration of virus material using small scale techniques such as g-force step-gradient ultracentrifugation or via conventional downstream processing methods, for instance crossflow filtration are crucial for more comprehensive site-specific *N*-glycosylation analysis of the ZIKV E protein and viral proteins in general in the future.

## Supplementary Information

Supplementary Informations.

## Data Availability

The datasets generated and analyzed in the scope of this study are available from the corresponding author upon request.

## Abbreviations

ACN: Acetonitril
ABC: Ammonium bicarbonate
AspN: Endoproteinase AspN
DTT: DL-dithiothreitol
E protein: Envelope protein
FASP: Filter-aided sample preparation
GlcNAc: *N*-Acetylglucosamine
HCD: Higher-energy collisional dissociation
HexNAc: *N*-Acetylhexosamine
HILIC: Hydrophilic interaction liquid chromatography
IAA: Iodoacetamide
LC: Liquid chromatography
nanoRP-LC: Nano reversed-phase liquid chromatography
M protein: Membrane protein
Man: Mannose
MOI: Multiplicity of infection
MS: Mass spectrometry
MS/MS: Tandem mass spectrometry
MWCO: Molecular weight cut-off
NS1: Non-structural protein 1
PBS: Phosphate-buffered saline
PCR: Polymerase chain reaction
PDB: Protein data bank
PNGase F: Peptide *N*-glycosidase F
prM: Precursor membrane protein
RP: Reversed-phase
SPE: Solid phase extraction
TBK1: Tank-binding kinase 1
ZIKV: Zika virus
