# Supplementary material for: Site-specific N-glycosylation analysis of animal cell culture-derived Zika virus proteins
Source: Sci Rep. 2021 Mar 4;11:5147. doi: 10.1038/s41598-021-84682-z (PMC7933209; doi:10.1038/s41598-021-84682-z)
Supplement: Supplementary file 1 — Supplementary Informations. [file 41598_2021_84682_MOESM1_ESM.docx]

Site-specific *N*-glycosylation analysis of animal cell culture-derived Zika virus proteins

Alexander Pralow ^1^, Alexander Nikolay ^1^, Arnaud Leon ^2^, Yvonne Genzel ^1^, Erdmann Rapp ^1, 3,^*, Udo Reichl ^1, 4^

^1^ Max Planck Institute for Dynamics of Complex Technical Systems, Bioprocess Engineering Group, Magdeburg, Germany

^2^ Valneva SE, Saint-Herblain, France

^3^ glyXera GmbH, Magdeburg, Germany

^4^ Otto von Guericke University, Chair of Bioprocess Engineering, Magdeburg, Germany

# SUPPLEMENT

# Annotated *N*-glycopeptide spectra

Amino acids indicated in red are potentially glycosylated amino acids.

Red peaks are oxonium ions.

Blue peaks are ions from the peptide backbone.

Green peaks indicate the intact peptide + HexNAc, peptide + 83 Da, peptide and peptide –NH_3_ pattern ^23^.

### Trypsin digest


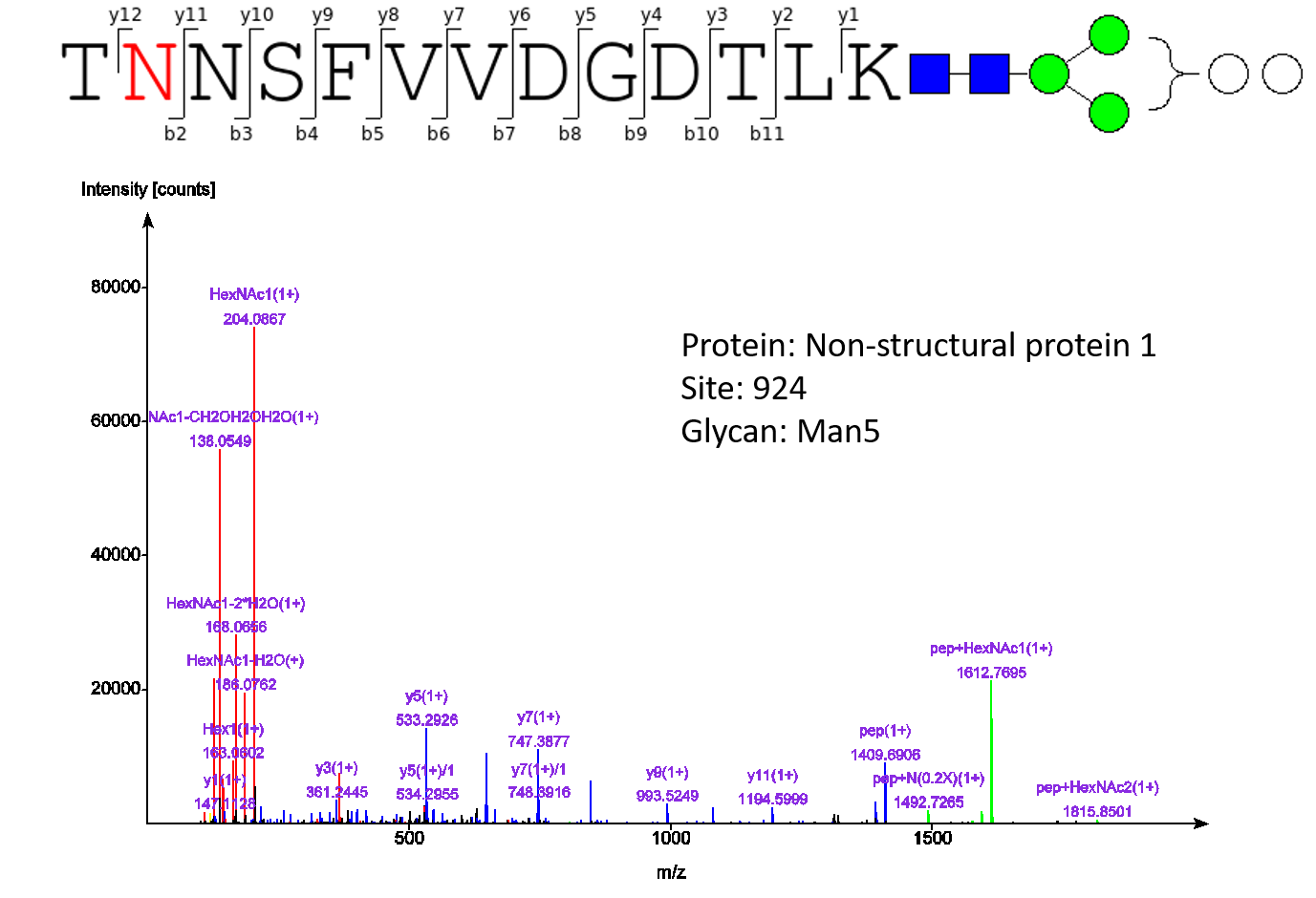

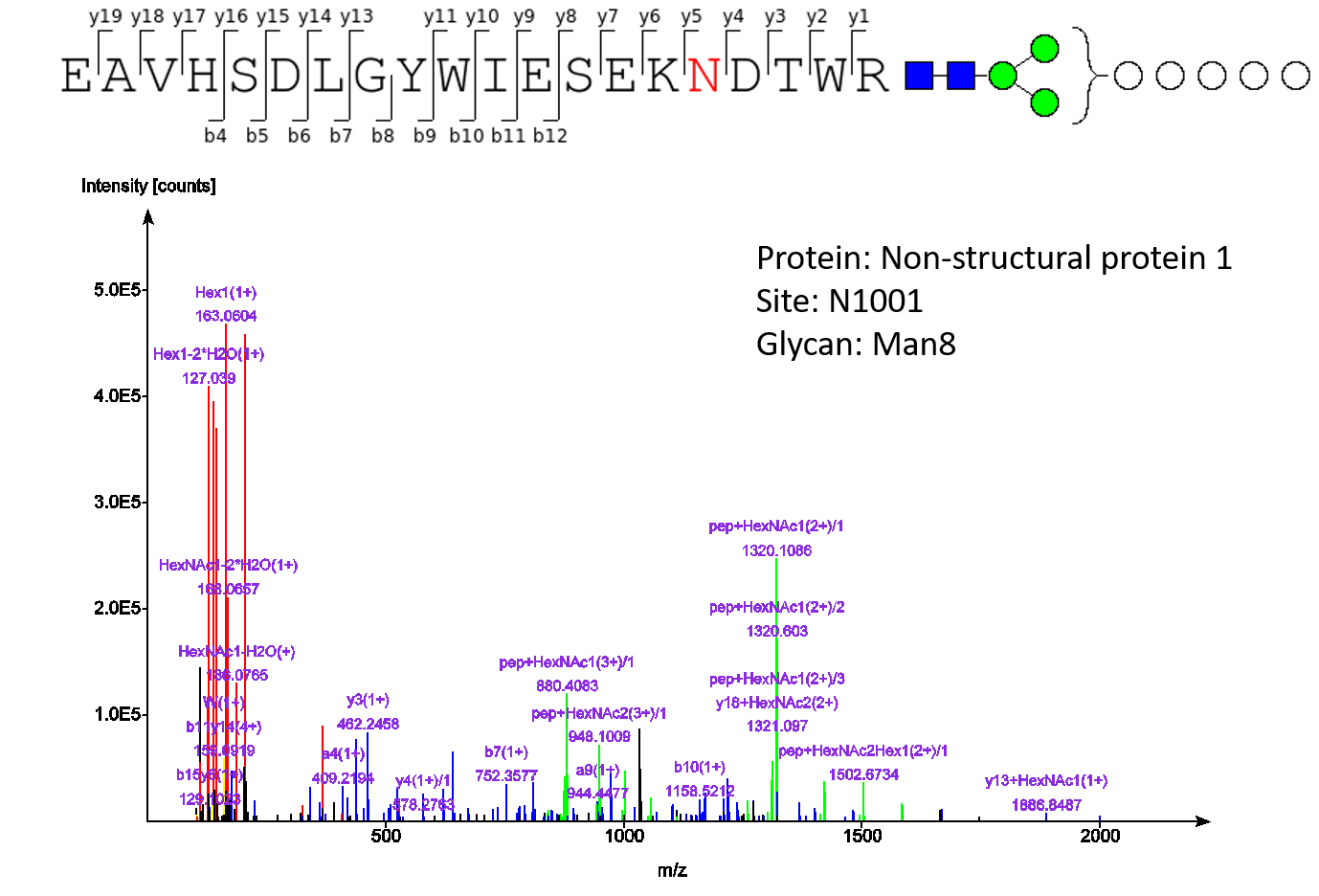


### Proteinase K digest


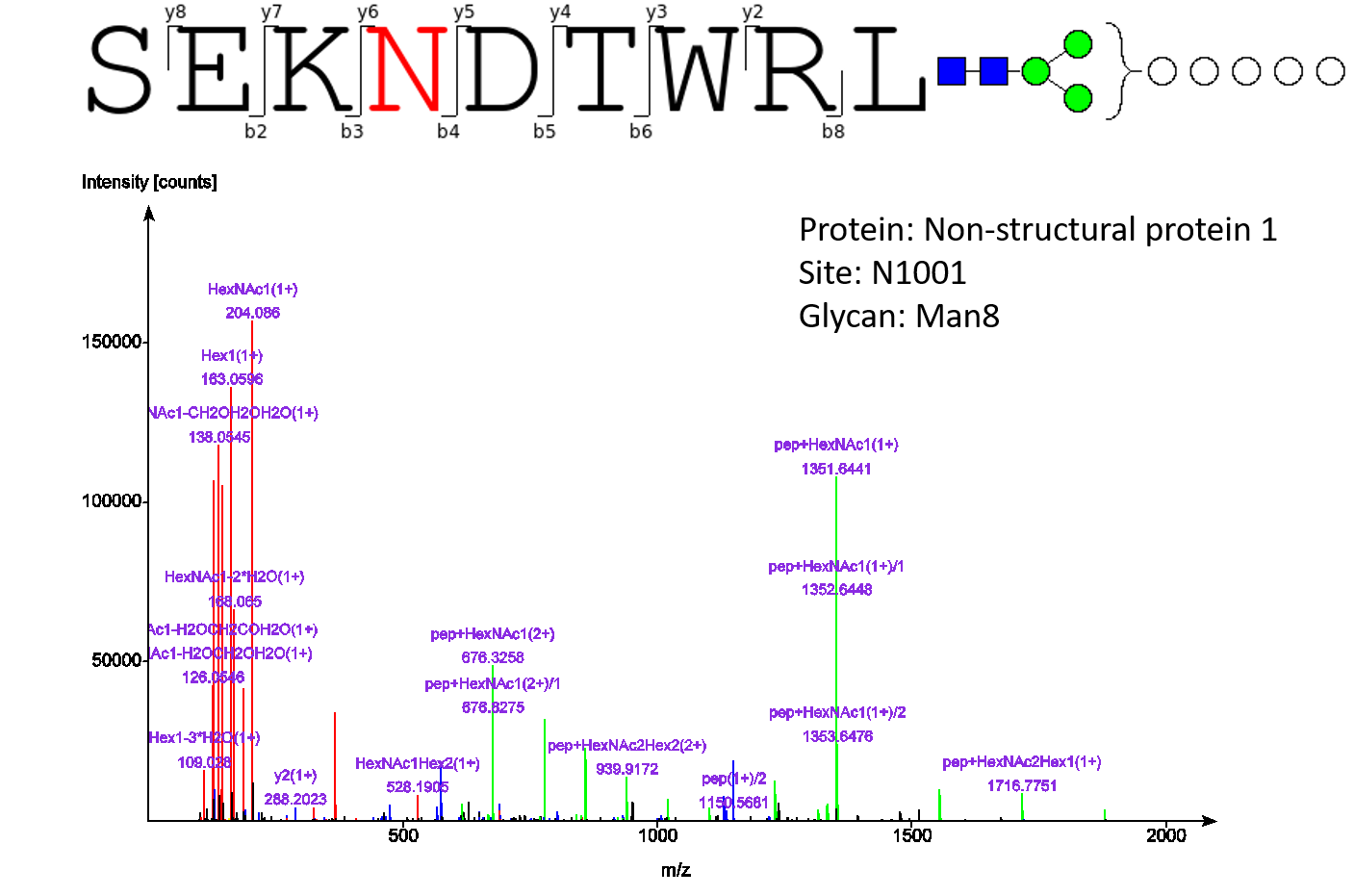


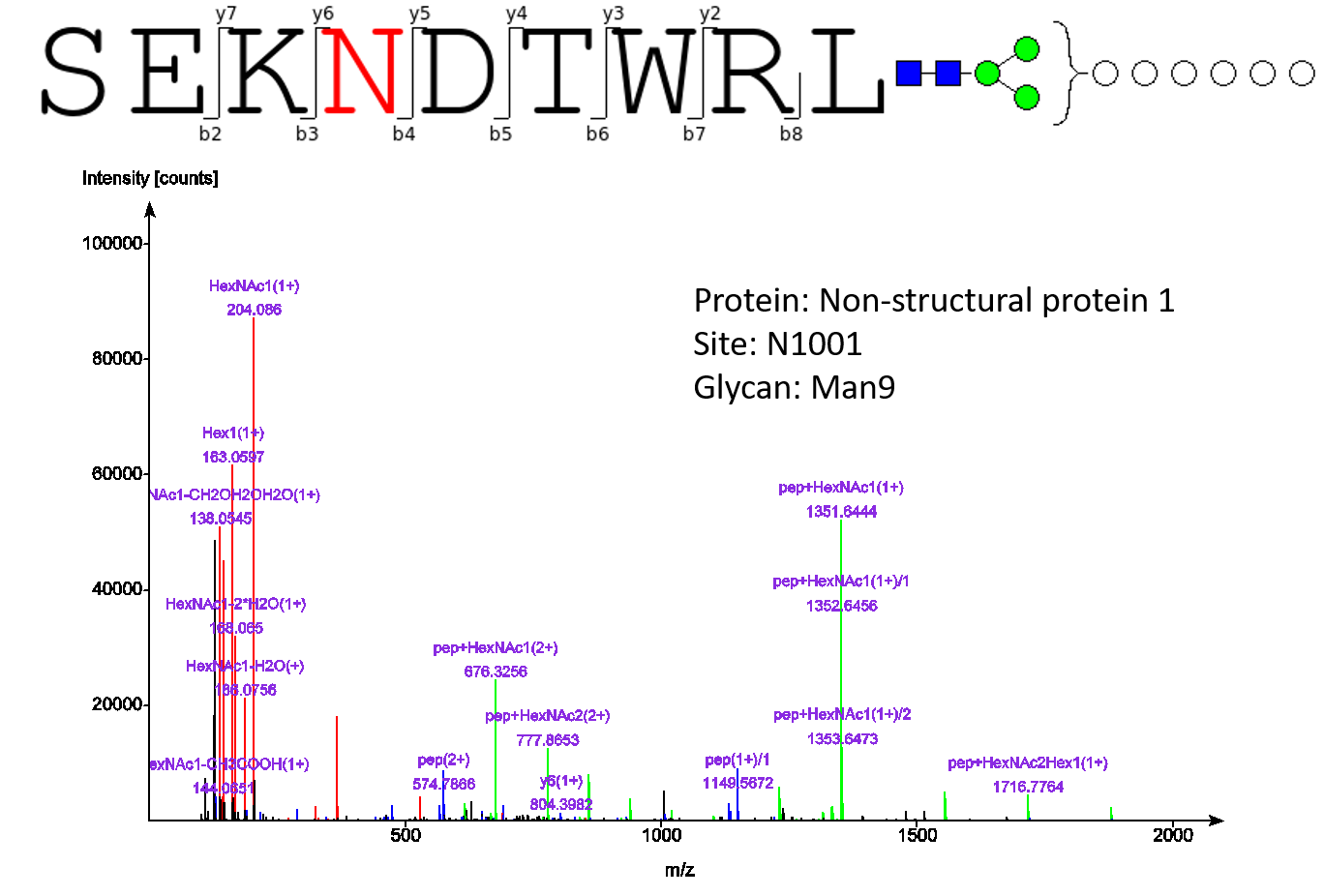


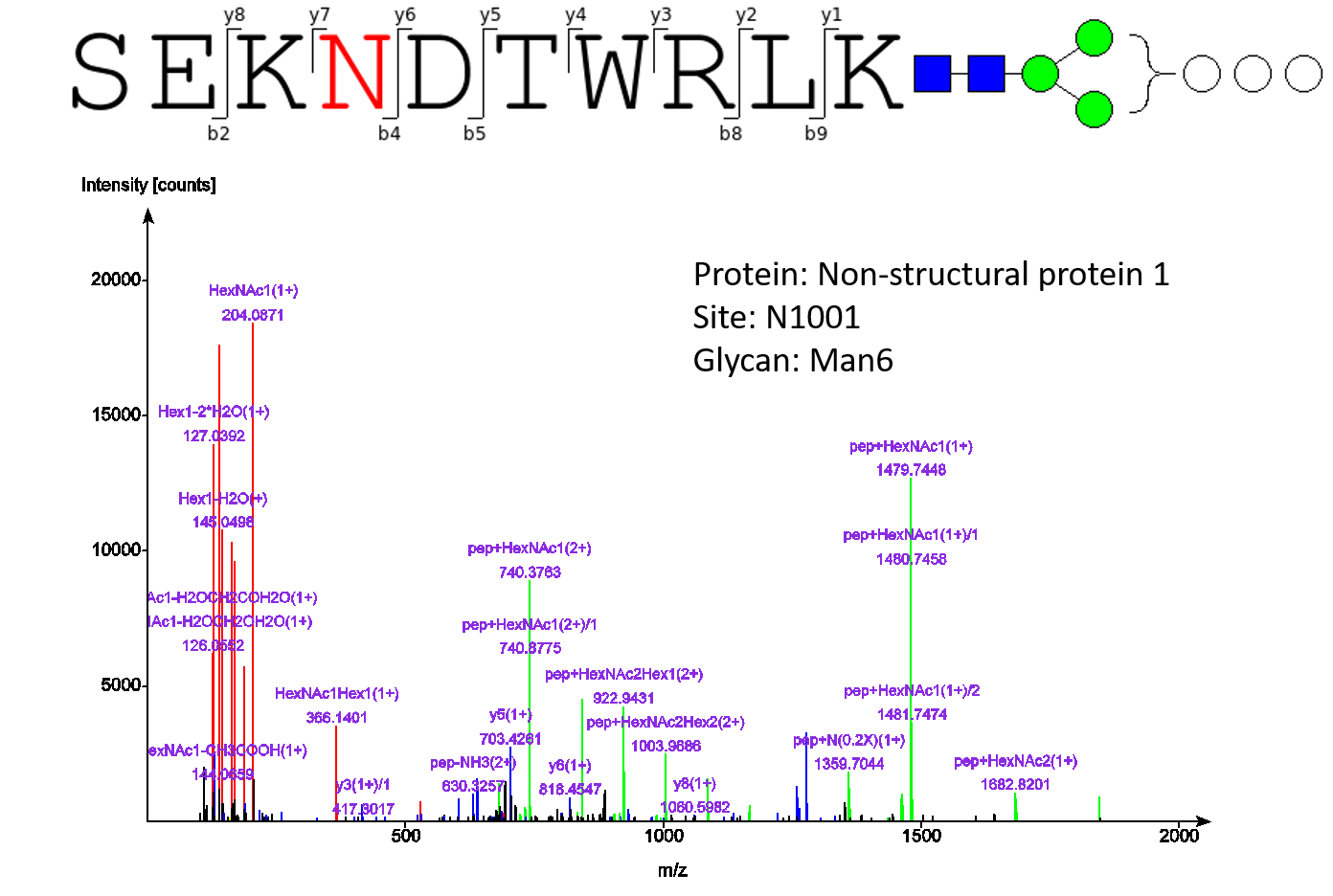


### Sequential Digest (Trypsin and Flavastacin)


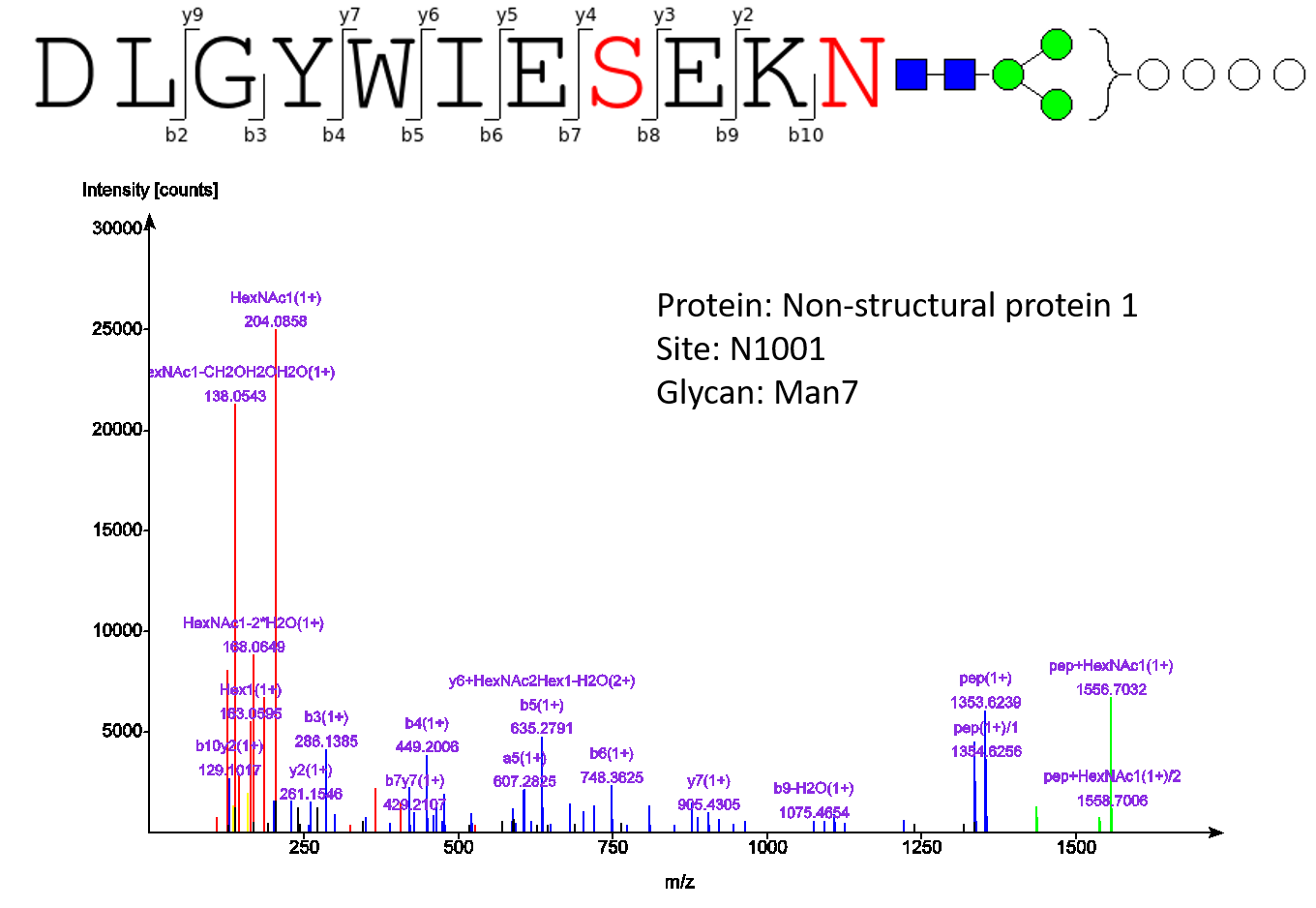


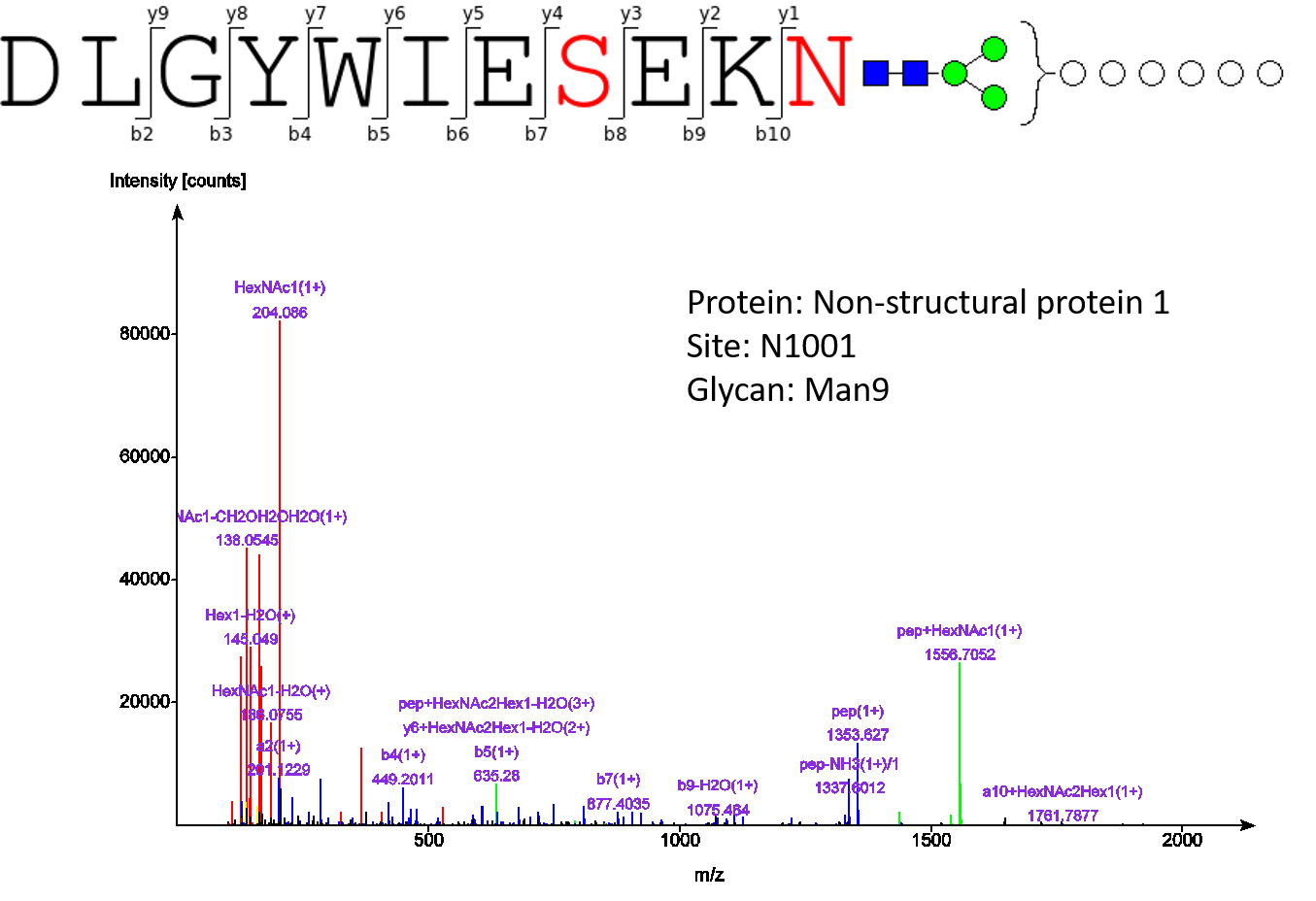


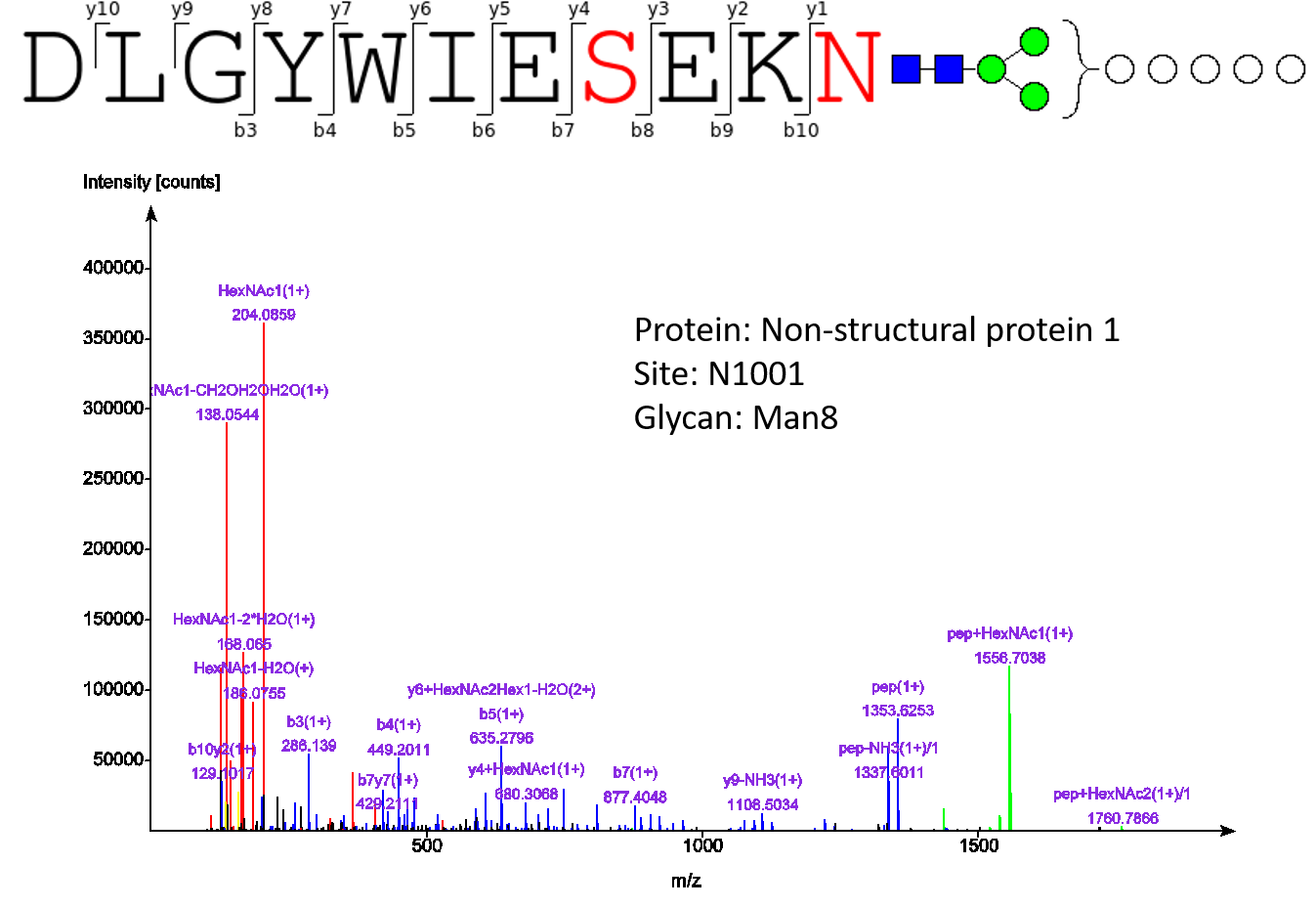


# E protein sequence coverage


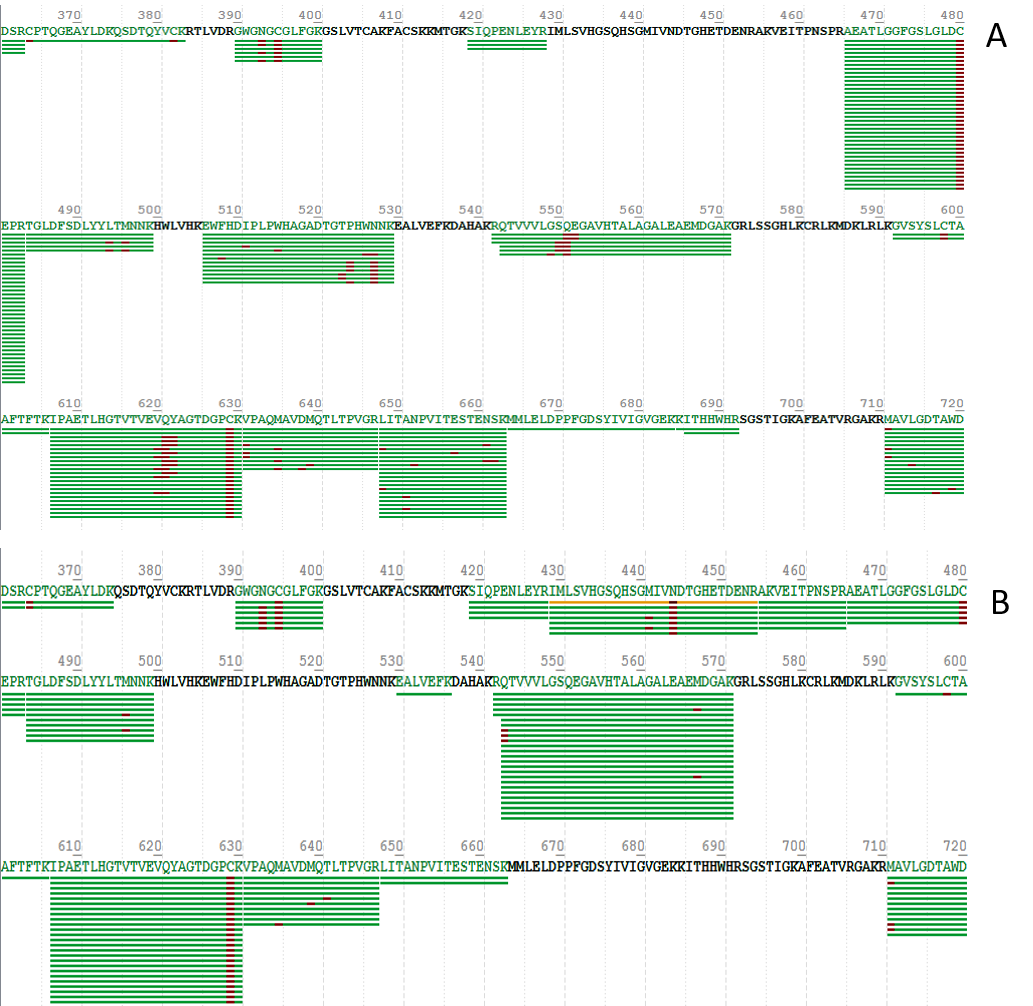


Supplement, Figure 1: Sequence coverage of tryptic digested ZIKV E protein produced in EB66 cells before (A) and after (B) N-glycan release with PNGaseF. Peptide sequence of the potential N-glycosylation site (N444) could be identified with a deamidation of asparagine to aspartic acid due to N-glycan release via PNGase F treatment. The green bars indicate individual MS/MS fragment ion spectra for the peptide sequence above respectively. Red marks indicate post-translational modifications. The orange bar shows the MS/MS fragment ion spectrum used in figure 3. The red circle highlights the potential N-glycopeptide sequence of the E protein. Sequence coverage based on UniProtKB A0A024B7W1 position 291-794 was 54% before and 50% after PNGaseF treatment.

# Proteomic analysis of enriched virus particles after ultracentrifugation

Table 1: Proteomic analysis of ZIKV. ZIKV was enriched via ultracentrifugation and inactivated using SDS. Proteins were digested using trypsin, measured via LC-MS and analyzed using Proteome Discoverer (Thermo Fisher Scientific).

| UniProt ID | Protein | Sequence Coverage | Unique Peptides | Peptides |
| --- | --- | --- | --- | --- |
| A0A024B7W1 | Genome polyprotein OS=Zika virus (isolate ZIKV/Human/French Polynesia/10087PF/2013) OX=2043570 PE=1 SV=1 - [POLG_ZIKVF] | 14.69 % | 1 | 39 |
| P11442 | Clathrin heavy chain 1 OS=Rattus norvegicus GN=Cltc PE=1 SV=3 - [CLH1_RAT] | 26.51 % | 4 | 29 |
| P11501 | Heat shock protein HSP 90-alpha OS=Gallus gallus GN=HSP90AA1 PE=3 SV=3 - [HS90A_CHICK] | 29.81 % | 1 | 15 |
| Q2KJD0 | Tubulin beta-5 chain OS=Bos taurus GN=TUBB5 PE=2 SV=1 - [TBB5_BOVIN] | 58.11 % | 2 | 5 |
| Q3MHM5 | Tubulin beta-4B chain OS=Bos taurus GN=TUBB4B PE=2 SV=1 - [TBB4B_BOVIN] | 51.24 % | 1 | 3 |
| P84856 | Actin, cytoplasmic 1 OS=Cercopithecus pygerythrus GN=ACTB PE=1 SV=1 - [ACTB_CERPY] | 53.74 % | 12 | 12 |
| P81947 | Tubulin alpha-1B chain OS=Bos taurus PE=1 SV=2 - [TBA1B_BOVIN] | 48.34 % | 2 | 11 |
| Q90835 | Elongation factor 1-alpha 1 OS=Gallus gallus GN=EEF1A PE=2 SV=1 - [EF1A_CHICK] | 41.34 % | 1 | 10 |
| P38650 | Cytoplasmic dynein 1 heavy chain 1 OS=Rattus norvegicus GN=Dync1h1 PE=1 SV=1 - [DYHC1_RAT] | 3.21 % | 2 | 8 |
| Q90705 | Elongation factor 2 OS=Gallus gallus GN=EEF2 PE=1 SV=3 - [EF2_CHICK] | 17.37 % | 1 | 8 |
| P00548 | Pyruvate kinase PKM OS=Gallus gallus GN=PKM PE=2 SV=2 - [KPYM_CHICK] | 22.64 % | 1 | 8 |
| P14105 | Myosin-9 OS=Gallus gallus GN=MYH9 PE=2 SV=1 - [MYH9_CHICK] | 6.18 % | 1 | 8 |
| A6YRY8 | 40S ribosomal protein SA OS=Ovis aries GN=RPSA PE=2 SV=1 - [RSSA_SHEEP] | 43.73 % | 10 | 8 |
| Q5ZJK8 | T-complex protein 1 subunit eta OS=Gallus gallus GN=CCT7 PE=1 SV=1 - [TCPH_CHICK] | 22.06 % | 1 | 8 |
| O57479 | Glyceraldehyde-3-phosphate dehydrogenase OS=Columba livia GN=GAPDH PE=2 SV=3 - [G3P_COLLI] | 32.13 % | 1 | 7 |
| E2RH47 | 40S ribosomal protein S3 OS=Canis familiaris GN=RPS3 PE=1 SV=1 - [RS3_CANFA] | 36.63 % | 3 | 7 |
| P80314 | T-complex protein 1 subunit beta OS=Mus musculus GN=Cct2 PE=1 SV=4 - [TCPB_MOUSE] | 18.50 % | 3 | 7 |
| P35489 | Dihydrolipoyllysine-residue acetyltransferase component of pyruvate dehydrogenase complex OS=Acholeplasma laidlawii GN=pdhC PE=1 SV=1 - [ODP2_ACHLA] | 16.18 % | 1 | 6 |
| P08267 | Ferritin heavy chain OS=Gallus gallus GN=FTH PE=2 SV=2 - [FRIH_CHICK] | 34.44 % | 1 | 5 |
| P63243 | Guanine nucleotide-binding protein subunit beta-2-like 1 OS=Bos taurus GN=GNB2L1 PE=2 SV=3 - [GBLP_BOVIN] | 32.49 % | 1 | 5 |
| P15771 | Nucleolin OS=Gallus gallus GN=NCL PE=1 SV=1 - [NUCL_CHICK] | 10.81 % | 1 | 5 |
| Q6EE31 | T-complex protein 1 subunit theta OS=Gallus gallus GN=CCT8 PE=1 SV=3 - [TCPQ_CHICK] | 12.77 % | 1 | 5 |
| P19140 | Alpha-enolase OS=Anas platyrhynchos GN=ENO1 PE=2 SV=2 - [ENOA_ANAPL] | 14.98 % | 1 | 4 |
| P08436 | Histone H4 OS=Volvox carteri GN=H4-I PE=3 SV=2 - [H4_VOLCA] | 33.98 % | 18 | 4 |
| O73885 | Heat shock cognate 71 kDa protein OS=Gallus gallus GN=HSPA8 PE=1 SV=1 - [HSP7C_CHICK] | 8.36 % | 1 | 4 |
| P35488 | Pyruvate dehydrogenase E1 component subunit beta OS=Acholeplasma laidlawii GN=pdhB PE=1 SV=1 - [ODPB_ACHLA] | 20.18 % | 1 | 4 |
| P51903 | Phosphoglycerate kinase OS=Gallus gallus GN=PGK PE=2 SV=2 - [PGK_CHICK] | 15.59 % | 1 | 4 |
| P12970 | 60S ribosomal protein L7a OS=Mus musculus GN=Rpl7a PE=2 SV=2 - [RL7A_MOUSE] | 19.92 % | 3 | 4 |
| Q29308 | 40S ribosomal protein S19 (Fragment) OS=Sus scrofa GN=RPS19 PE=2 SV=3 - [RS19_PIG] | 23.53 % | 3 | 4 |
| O43999 | 40S ribosomal protein S3a OS=Eimeria tenella PE=2 SV=3 - [RS3A_EIMTE] | 25.00 % | 1 | 4 |
| Q9W790 | T-complex protein 1 subunit alpha OS=Paleosuchus palpebrosus GN=TCP1 PE=2 SV=1 - [TCPA_PALPA] | 13.42 % | 1 | 4 |
| P50991 | T-complex protein 1 subunit delta OS=Homo sapiens GN=CCT4 PE=1 SV=4 - [TCPD_HUMAN] | 10.20 % | 4 | 4 |
| P49368 | T-complex protein 1 subunit gamma OS=Homo sapiens GN=CCT3 PE=1 SV=4 - [TCPG_HUMAN] | 10.09 % | 6 | 4 |
| Q5ZJ54 | T-complex protein 1 subunit zeta OS=Gallus gallus GN=CCT6 PE=1 SV=3 - [TCPZ_CHICK] | 14.15 % | 1 | 4 |
| Q06066 | Nuclease-sensitive element-binding protein 1 OS=Gallus gallus GN=YBX1 PE=2 SV=1 - [YBOX1_CHICK] | 28.97 % | 1 | 4 |
| P69069 | Histone H2B OS=Oncorhynchus mykiss PE=1 SV=2 - [H2B_ONCMY] | 28.23 % | 32 | 3 |
| Q7TP47 | Heterogeneous nuclear ribonucleoprotein Q OS=Rattus norvegicus GN=Syncrip PE=2 SV=1 - [HNRPQ_RAT] | 6.75 % | 3 | 3 |
| Q5ZLP8 | Insulin-like growth factor 2 mRNA-binding protein 3 OS=Gallus gallus GN=IGF2BP3 PE=2 SV=1 - [IF2B3_CHICK] | 9.25 % | 1 | 3 |
| Q3SZ65 | Eukaryotic initiation factor 4A-II OS=Bos taurus GN=EIF4A2 PE=2 SV=1 - [IF4A2_BOVIN] | 9.34 % | 3 | 3 |
| P56701 | 26S proteasome non-ATPase regulatory subunit 2 OS=Bos taurus GN=PSMD2 PE=1 SV=2 - [PSMD2_BOVIN] | 5.95 % | 5 | 3 |
| P21531 | 60S ribosomal protein L3 OS=Rattus norvegicus GN=Rpl3 PE=1 SV=3 - [RL3_RAT] | 11.66 % | 3 | 3 |
| Q9DG68 | 60S acidic ribosomal protein P0 OS=Rana sylvatica GN=RPLP0 PE=2 SV=1 - [RLA0_RANSY] | 13.97 % | 2 | 3 |
| P49393 | 40S ribosomal protein S13 OS=Xenopus laevis GN=rps13 PE=3 SV=2 - [RS13_XENLA] | 19.87 % | 4 | 3 |
| P62983 | Ubiquitin-40S ribosomal protein S27a OS=Mus musculus GN=Rps27a PE=2 SV=2 - [RS27A_MOUSE] | 24.36 % | 4 | 3 |
| O62739 | 40S ribosomal protein S4, Y isoform 1 OS=Monodelphis domestica GN=RPS4Y1 PE=2 SV=3 - [RS4Y1_MONDO] | 13.69 % | 7 | 3 |
| Q5E988 | 40S ribosomal protein S5 OS=Bos taurus GN=RPS5 PE=2 SV=3 - [RS5_BOVIN] | 23.53 % | 1 | 3 |
| P00761 | Trypsin OS=Sus scrofa PE=1 SV=1 - [TRYP_PIG] | 20.78 % | 1 | 3 |
| Q5ZLQ6 | 14-3-3 protein beta/alpha OS=Gallus gallus GN=YWHAB PE=2 SV=1 - [1433B_CHICK] | 11.48 % | 3 | 2 |
| P30153 | Serine/threonine-protein phosphatase 2A 65 kDa regulatory subunit A alpha isoform OS=Homo sapiens GN=PPP2R1A PE=1 SV=4 - [2AAA_HUMAN] | 5.94 % | 7 | 2 |
| P17427 | AP-2 complex subunit alpha-2 OS=Mus musculus GN=Ap2a2 PE=1 SV=2 - [AP2A2_MOUSE] | 4.37 % | 3 | 2 |
| P23220 | Plasma membrane calcium-transporting ATPase 1 OS=Sus scrofa GN=ATP2B1 PE=2 SV=1 - [AT2B1_PIG] | 2.54 % | 2 | 2 |
| P26231 | Catenin alpha-1 OS=Mus musculus GN=Ctnna1 PE=1 SV=1 - [CTNA1_MOUSE] | 5.19 % | 4 | 2 |
| P18359 | Destrin OS=Gallus gallus GN=DSTN PE=1 SV=3 - [DEST_CHICK] | 16.36 % | 1 | 2 |
| Q13838 | Spliceosome RNA helicase DDX39B OS=Homo sapiens GN=DDX39B PE=1 SV=1 - [DX39B_HUMAN] | 7.01 % | 5 | 2 |
| Q8CGP6 | Histone H2A type 1-H OS=Mus musculus GN=Hist1h2ah PE=1 SV=3 - [H2A1H_MOUSE] | 21.88 % | 23 | 2 |
| Q5ZIQ3 | Heterogeneous nuclear ribonucleoprotein K OS=Gallus gallus GN=HNRNPK PE=2 SV=1 - [HNRPK_CHICK] | 6.79 % | 5 | 2 |
| P10160 | Eukaryotic translation initiation factor 5A-1 OS=Oryctolagus cuniculus GN=EIF5A PE=1 SV=2 - [IF5A1_RABIT] | 22.73 % | 2 | 2 |
| Q5ZMI4 | Major vault protein OS=Gallus gallus GN=MVP PE=2 SV=1 - [MVP_CHICK] | 5.18 % | 1 | 2 |
| Q6IP73 | Nascent polypeptide-associated complex subunit alpha OS=Xenopus laevis GN=naca PE=2 SV=1 - [NACA_XENLA] | 12.68 % | 10 | 2 |
| Q61990 | Poly(rC)-binding protein 2 OS=Mus musculus GN=Pcbp2 PE=1 SV=1 - [PCBP2_MOUSE] | 12.98 % | 2 | 2 |
| P09102 | Protein disulfide-isomerase OS=Gallus gallus GN=P4HB PE=1 SV=3 - [PDIA1_CHICK] | 4.85 % | 1 | 2 |
| P62191 | 26S protease regulatory subunit 4 OS=Homo sapiens GN=PSMC1 PE=1 SV=1 - [PRS4_HUMAN] | 7.95 % | 2 | 2 |
| P54775 | 26S protease regulatory subunit 6B OS=Mus musculus GN=Psmc4 PE=1 SV=2 - [PRS6B_MOUSE] | 9.09 % | 2 | 2 |
| P46471 | 26S protease regulatory subunit 7 OS=Mus musculus GN=Psmc2 PE=1 SV=5 - [PRS7_MOUSE] | 6.47 % | 5 | 2 |
| P62194 | 26S protease regulatory subunit 8 OS=Bos taurus GN=PSMC5 PE=2 SV=1 - [PRS8_BOVIN] | 9.11 % | 2 | 2 |
| B0BN93 | 26S proteasome non-ATPase regulatory subunit 13 OS=Rattus norvegicus GN=Psmd13 PE=1 SV=1 - [PSD13_RAT] | 10.90 % | 4 | 2 |
| P26516 | 26S proteasome non-ATPase regulatory subunit 7 OS=Mus musculus GN=Psmd7 PE=1 SV=2 - [PSMD7_MOUSE] | 7.17 % | 3 | 2 |
| Q06AU7 | Ras-related protein Rab-1B OS=Sus scrofa GN=RAB1B PE=2 SV=1 - [RAB1B_PIG] | 13.43 % | 9 | 2 |
| P80236 | Ras-related C3 botulinum toxin substrate 1 (Fragment) OS=Cavia porcellus GN=RAC1 PE=1 SV=2 - [RAC1_CAVPO] | 31.17 % | 2 | 2 |
| O17915 | GTP-binding nuclear protein ran-1 OS=Caenorhabditis elegans GN=ran-1 PE=1 SV=1 - [RAN_CAEEL] | 11.63 % | 7 | 2 |
| P23358 | 60S ribosomal protein L12 OS=Rattus norvegicus GN=Rpl12 PE=2 SV=1 - [RL12_RAT] | 14.55 % | 4 | 2 |
| P79324 | 60S ribosomal protein L15 (Fragment) OS=Sus scrofa GN=RPL15 PE=2 SV=1 - [RL15_PIG] | 16.99 % | 3 | 2 |
| P67883 | 60S ribosomal protein L30 OS=Gallus gallus GN=RPL30 PE=3 SV=2 - [RL30_CHICK] | 26.96 % | 3 | 2 |
| Q9D8E6 | 60S ribosomal protein L4 OS=Mus musculus GN=Rpl4 PE=1 SV=3 - [RL4_MOUSE] | 9.79 % | 6 | 2 |
| Q3T0S6 | 60S ribosomal protein L8 OS=Bos taurus GN=RPL8 PE=2 SV=3 - [RL8_BOVIN] | 17.90 % | 3 | 2 |
| Q07254 | 40S ribosomal protein S10 OS=Xenopus laevis GN=rps10 PE=1 SV=1 - [RS10_XENLA] | 14.55 % | 2 | 2 |
| Q3T0V4 | 40S ribosomal protein S11 OS=Bos taurus GN=RPS11 PE=2 SV=3 - [RS11_BOVIN] | 16.46 % | 1 | 2 |
| Q5R938 | 40S ribosomal protein S15a OS=Pongo abelii GN=RPS15A PE=2 SV=1 - [RS15A_PONAB] | 17.69 % | 3 | 2 |
| Q3T0X6 | 40S ribosomal protein S16 OS=Bos taurus GN=RPS16 PE=2 SV=3 - [RS16_BOVIN] | 14.38 % | 3 | 2 |
| Q90YQ6 | 40S ribosomal protein S17 OS=Ictalurus punctatus GN=rps17 PE=2 SV=3 - [RS17_ICTPU] | 25.37 % | 5 | 2 |
| Q3T0R1 | 40S ribosomal protein S18 OS=Bos taurus GN=RPS18 PE=2 SV=3 - [RS18_BOVIN] | 13.82 % | 3 | 2 |
| Q3T199 | 40S ribosomal protein S23 OS=Bos taurus GN=RPS23 PE=2 SV=1 - [RS23_BOVIN] | 26.57 % | 3 | 2 |
| Q56JX5 | 40S ribosomal protein S25 OS=Bos taurus GN=RPS25 PE=2 SV=1 - [RS25_BOVIN] | 14.40 % | 1 | 2 |
| P46791 | 40S ribosomal protein S2 (Fragment) OS=Cricetulus griseus GN=RPS2 PE=2 SV=1 - [RS2_CRIGR] | 14.85 % | 4 | 2 |
| A6H769 | 40S ribosomal protein S7 OS=Bos taurus GN=RPS7 PE=2 SV=1 - [RS7_BOVIN] | 17.53 % | 1 | 2 |
| Q29197 | 40S ribosomal protein S9 (Fragment) OS=Sus scrofa GN=RPS9 PE=2 SV=1 - [RS9_PIG] | 15.38 % | 2 | 2 |
| A5A6P1 | D-3-phosphoglycerate dehydrogenase OS=Pan troglodytes GN=PHGDH PE=2 SV=1 - [SERA_PANTR] | 4.32 % | 7 | 2 |
| P49591 | Serine--tRNA ligase, cytoplasmic OS=Homo sapiens GN=SARS PE=1 SV=3 - [SYSC_HUMAN] | 4.47 % | 3 | 2 |
| P48643 | T-complex protein 1 subunit epsilon OS=Homo sapiens GN=CCT5 PE=1 SV=1 - [TCPE_HUMAN] | 5.73 % | 4 | 2 |
